# Research progress on the effects of M1/M2 macrophages on the differentiation and maturation of stem cell-derived cardiomyocytes: a review

**DOI:** 10.1186/s13287-026-04938-1

**Published:** 2026-02-15

**Authors:** Xi Wu, Fan Zhou, Junsheng Mu

**Affiliations:** 1 Department of Cardiac Surgery, The Third Affiliated Hospital of Xinxiang Medical University, Xinxiang, 453000 Henan China; 2 Department of Ultrasound, The Third Medical Center of People’s Liberation Army of China General Hospital, Beijing, 100039 China; 3 Department of Cardiac Surgery, Beijing Anzhen Hospital, Beijing Institute of Heart Lung and Blood Vessel Diseases, Capital Medical University, Beijing, 100029 China

**Keywords:** Macrophage polarization, Stem cell-derived cardiomyocytes, Maturation, Tissue regeneration, Immune microenvironment

## Abstract

Stem cell-derived cardiomyocytes (SC-CMs) represent a promising cell source for cardiac regenerative medicine, disease modeling, and drug screening. However, their clinical translation faces significant challenges, including functional immaturity, poor long-term survival, and inadequate integration with host tissue following transplantation. The immune microenvironment, particularly the dynamic polarization of macrophages into pro-inflammatory (M1) or reparative (M2) phenotypes, is increasingly recognized as a critical regulator of cardiac repair, yet a systematic understanding of its specific effects on SC-CM fate remains incomplete. This review aims to comprehensively evaluate the dual regulatory roles of M1 and M2 macrophages on the differentiation efficiency, structural and functional maturation, and in vivo transplantation efficacy of SC-CMs. A systematic literature search was conducted in PubMed, Web of Science, Nature, and CNKI for relevant studies published from database inception to July 2025. After screening, 92 articles were included for analysis. The synthesized evidence demonstrates that M1 macrophages and their secreted factors (e.g., TNF-α, IL-1β) impede cardiac differentiation by inhibiting the Wnt/β-catenin pathway, disrupt sarcomeric organization and calcium handling, and maintain SC-CMs in a glycolytic, immature state. In contrast, M2 macrophages enhance SC-CM maturation by providing trophic support (e.g., IGF-1, HGF), promoting electrophysiological maturation and metabolic reprogramming towards oxidative phosphorylation, and facilitating angiogenesis via VEGF. The novelty of this review lies in its integrated perspective on macrophage-driven immunomodulation as a central axis for SC-CM maturation. Furthermore, it discusses emerging therapeutic strategies—such as optimized transplantation timing, co-transplantation with immunomodulatory cells, engineered exosomes, and smart biomaterials—that leverage macrophage polarization to create a favorable microenvironment for SC-CMs. Ultimately, harnessing macrophage-SC-CM crosstalk is a crucial step toward advancing clinically effective and immunologically informed cardiac regeneration therapies.

## Background

Cardiovascular disease is the leading cause of death worldwide. Myocardial infarction (MI), which leads to massive loss of cardiomyocytes and subsequent fibrotic scar formation, is a major pathological basis for heart failure [1]. The self-regenerative capacity of the adult heart is extremely limited. Therefore, treatment for end-stage heart failure often ultimately relies on heart transplantation, but the severe shortage of donors limits this approach [2]. The rise of regenerative medicine, particularly the development of human pluripotent stem cell (hPSC) technologies, including embryonic stem cells and induced pluripotent stem cells, provides an unlimited cell source for generating functional cardiomyocytes (i.e., stem cell-derived cardiomyocytes [SC-CMs]), offering revolutionary hope for cardiac repair [3].

However, despite technological advancements, the clinical application of SC-CMs still faces two core challenges. First, SC-CMs obtained from in vitro differentiation resemble fetal rather than adult cardiomyocytes in terms of structure, gene expression, metabolism, and electrophysiological properties. This functional immaturity of SC-CMs causes difficulty for them to achieve effective electro-mechanical integration with host mature myocardium and may even trigger fatal arrhythmias [4]. Second, when SC-CMs are transplanted into the injured heart, they encounter an extremely hostile microenvironment, such as ischemia, hypoxia, and a strong host immune inflammatory response, leading to massive death of the transplanted cells. Additionally, tumorigenicity is a major concern in the application of pluripotent stem cells (PSCs) for myocardial regeneration because cardiac regeneration requires large quantities of cardiomyocytes differentiated from PSCs [5].

Previously, the heart was often considered a terminally differentiated organ with few immune cells. Traditional research primarily focused on optimizing biochemical signaling pathways (e.g., BMP, Wnt, and FGF) for inducing cardiac differentiation [6]. However, increasing evidence in recent years has indicated that the heart possesses a unique immune ecosystem that maintains a state of “vigilant balance” under homeostasis. This ecosystem is capable of responding to minor injury while avoiding excessive inflammatory reactions that damage irreplaceable cardiomyocytes. This delicate balance can be disrupted, shifting the response from protective repair to destructive inflammation. When tissue damage and stress occur, numerous damage-associated molecular patterns (DAMPs) are released, activating pattern recognition receptors (e.g., Toll-like receptors) and triggering a robust infiltration of neutrophils and monocytes/macrophages. The microenvironment rapidly shifts from an anti-inflammatory state to a strongly pro-inflammatory state. Infiltrating monocytes differentiate into pro-inflammatory M1-type macrophages, with secretion of tumor necrosis factor (TNF)-α, interleukin (IL)-1β, and IL-6 to clear necrotic tissue. Ideally, the microenvironment transitions to a repair phase, with monocytes and resident macrophages shifting towards an M2 phenotype, promoting fibrotic scar formation. If inflammation fails to resolve in a timely manner, it leads to adverse remodeling and heart failure.

The cardiac immune microenvironment is primarily composed of core immune cell populations and their inherent immunity and physical barriers. The core cell populations include macrophages, T lymphocytes, neutrophils, dendritic cells, and mast cells. Among them, macrophages are the most abundant immune cells in the heart. These macrophages comprise tissue-resident macrophages, which migrate into the heart during embryonic development and maintain their population through self-proliferation, and monocyte-derived macrophages, which originate from the circulation and infiltrate in large numbers under inflammatory conditions. Macrophages clear senescent cellular debris (e.g., mitochondria), maintain tissue cleanliness through phagocytosis, act as the first line of defense by recognizing DAMPs, secrete growth factors to promote angiogenesis and matrix remodeling, and participate in the electrophysiological regulation of cardiomyocytes. Under homeostasis, the cardiac immune microenvironment, dominated by tissue-resident macrophages and regulatory T cells, maintains an immunosuppressive and tolerant state. The endogenous immune microenvironment plays a leading role in cardiac development, maintenance of homeostasis, and post-injury repair [7]. Following MI, dying cardiomyocytes release large amounts of DAMPs, which bind to various pattern recognition receptors, particularly Toll-like receptors, immediately initiating downstream signaling pathways (e.g., nuclear factor-κB and MAPK). This initiation leads to the rapid activation of cardiac resident macrophages and those recruited from monocytes. These macrophages polarize into different functional phenotypes according to local microenvironmental signals, transitioning from a predominance of the pro-inflammatory M1 type in the early phase of injury to the anti-inflammatory M2 type in the repair phase. This dynamic process precisely regulates the balance between inflammation resolution, matrix degradation, scar formation, and tissue remodeling [8] (Table 1).

**Table 1:** Core functional comparison between tissue-resident macrophages and recruited macrophages

Functional area	Cardiac resident macrophages	Recruited macrophages
Effect on cardiomyocyte apoptosis	**•** Under homeostasis: Directly inhibit cardiomyocyte apoptosis by secreting factors like Transforming Growth Factor-β (TGF-β), maintaining their survival. **•** Early injury: Function as scavengers, efficiently and quietly clearing apoptotic cell debris to prevent inflammation amplification. This process, called “efferocytosis”, can induce the production of anti-inflammatory factors like IL-10, promoting repair.	**•** During disease/inflammation: Primarily exhibit a pro-inflammatory (M1-like) phenotype. Secrete large amounts of pro-inflammatory factors like Tumor Necrosis Factor-α (TNF-α) and Interleukin-1β (IL-1β), which can directly induce or exacerbate cardiomyocyte apoptosis. **•** Late repair phase: Some may convert to an anti-inflammatory (M2-like) phenotype, secreting some protective factors, but their overall negative pro-apoptotic effect is more prominent.
Electrical signal integration and conduction	**•** Direct electrical coupling: Form direct electrochemical synapses with cardiomyocytes via gap junction channels like Connexin 43 (Cx43). **•** Function: (1) Electrical stability: May “absorb” some depolarizing current, increasing the electrical excitation threshold of cardiomyocytes, exerting an anti-arrhythmic effect. (2) Signal transmission: May participate in fine-tuning the cardiac conduction system, though specific mechanisms are still under exploration.	**•** Indirect electrical influence: Do not form direct gap junctions with cardiomyocytes. **•** Function: Primarily indirectly disrupt electrical signals through the secretion of pro-inflammatory factors (e.g., TNF-α, IL-1β). These factors alter the expression and function of ion channels (e.g., sodium, potassium, calcium channels) in cardiomyocytes, leading to prolonged action potential duration, slowed conduction velocity, and thus pro-arrhythmia.
“Relay” effect on other cells/signals	**•** Immune surveillance: Act as cardiac “sentinels,” sensing minor tissue damage or stress signals and coordinating initial immune responses via cytokine secretion. • Promoting repair: Promote angiogenesis by secreting growth factors (e.g., VEGF) and “clear the path” for tissue remodeling through their scavenging function.	• Inflammation amplification: After massive recruitment, amplify the inflammatory response via positive feedback by secreting potent chemokines, recruiting more immune cells (e.g., neutrophils, lymphocytes). • Fibrosis drive: Strongly activate cardiac fibroblasts by secreting factors like TGF-β and Platelet-Derived Growth Factor (PDGF), leading to pathological fibrosis, disrupting the synchrony of cardiac electro-mechanical activity.

Similar to the in vivo development and repair processes of the heart, the in vitro differentiation and maturation of SC-CMs, as well as their in vivo transplantation and integration, are likely also subject to precise regulation by immune signals [9].

Despite the established role of macrophages in cardiac wound healing, a systematic and critical appraisal of their direct and indirect impacts on the in vitro differentiation and in vivo maturation of SC-CMs is notably lacking. Previous reviews have often treated the immune response merely as a hostile barrier to cell transplantation or have provided a descriptive list of macrophage-secreted factors. The novelty of this review lies in its integrated framework that positions macrophage polarization as a central, dynamic regulator of SC-CM fate. We specifically address the knowledge gap concerning how the temporal switch from M1 to M2 polarization, which is crucial for in vivo repair, can be mimicked or harnessed to guide SC-CM maturation in vitro and improve their therapeutic integration in vivo. Furthermore, we critically evaluate the apparent contradictions in the literature, such as the context-dependent functions of certain cytokines, and discuss the translational challenges and prospects of combining SC-CM therapy with targeted immunomodulation strategies. This integrated perspective provides a novel roadmap for developing next-generation cardiac regenerative therapies that combine cell biology with precise immune engineering.

Therefore, systematically determining the effect of macrophage polarization on the fate of SC-CMs is not only of great theoretical importance but also provides novel insights and targets for developing next-generation regenerative therapies combined with immune regulation. This review summarizes the interactions between macrophages and SC-CMs, the underlying molecular mechanisms, and therapeutic strategies, and discusses future challenges and clinical application prospects.

## Biological basis of macrophage polarization

### Introduction

Currently, laboratory-differentiated hPSC-derived cardiomyocytes (SC-CMs) exhibit a fetal-like phenotype in structure, function, and metabolism, severely limiting their utility in disease modeling, drug screening, and cell therapy [10]. Mature adult cardiomyocytes, in contrast, are terminally differentiated cells characterized by: (1) a highly ordered sarcomeric structure and T-tubule system for efficient excitation–contraction coupling; (2) mature electrophysiological properties, including a stable resting membrane potential and adult-specific action potential morphology; (3) a metabolic shift from glycolysis to fatty acid β-oxidation as the primary energy source; and (4) strong contractility and cell cycle exit. SC-CMs display considerable limitations across all these aspects. Furthermore, current maturation strategies face several challenges: most approaches are isolated and one-sided, failing to recapitulate the synergistic multi-pathway action of in vivo maturation, which often results only in “partial” rather than true “adult-like” maturity. The field also lacks unified assessment standards, with different laboratories employing varied criteria for “maturation,” making cross-study comparisons difficult and raising the risk of “pseudo-maturation” where interventions alter specific phenotypes without enabling global functional maturation. Finally, combining multiple strategies (e.g., 3D culture, electrical stimulation, and metabolic regulation), while potentially more effective, increases system complexity, cost, and standardization difficulties, hindering large-scale application and clinical translation. Therefore, future research should prioritize developing integrated maturation platforms, deepening the understanding of underlying molecular mechanisms to identify fundamental targets, and establishing unified, rigorous assessment standards to advance the field.

### M1/M2 macrophages and their polarization spectrum

Macrophages originate from hematopoietic stem cells (HSCs) in the bone marrow, pass through the monocyte stage, and finally differentiate and reside in various tissues and organs. Macrophages are not a homogeneous cell population but possess high plasticity, allowing them to change their phenotype and function according to signals in their microenvironment, which is a process known as macrophage polarization [11]. Based on the activation mode and function of macrophages, they are mainly divided into two polarization phenotypes: the classically activated M1 type and the alternatively activated M2 type [12]. M1 macrophages are primarily activated by Th1 cytokines (e.g., interferon-γ), pathogen-associated molecular patterns ([PAMPs] e.g., lipopolysaccharide), or DAMPs. M1 macrophages highly express major histocompatibility complex class II molecules and co-stimulatory molecules (e.g., CD80 and CD86), and secrete large amounts of pro-inflammatory cytokines, such as TNF-α, (IL-1β, IL-6, IL-12, and IL-23. Simultaneously, M1 macrophages upregulate inducible nitric oxide synthase, producing large quantities of nitric oxide (NO) and reactive oxygen species (ROS), thereby effectively killing intracellular pathogens and clearing necrotic tissue [13]. Therefore, M1 macrophages are the dominant players in the acute inflammatory phase, but sustained or excessive M1 responses cause collateral tissue damage. In contrast, M2 macrophages are induced by signals, such as Th2 cytokines (e.g., IL-4 and IL-13), IL-10, glucocorticoids, and immune complexes. M2 macrophages highly express scavenger receptors (CD163 and CD206), the mannose receptor, and arginase 1. M2 macrophages do not produce high levels of pro-inflammatory cytokines but instead secrete large amounts of anti-inflammatory factors (e.g., IL-10 and transforming growth factor [TGF]-β) and various growth factors (e.g., vascular endothelial growth factor [VEGF], insulin-like growth factor insulin-like growth factor 1, and hepatocyte growth factor) [14]. The main functions of M2 macrophages are to suppress inflammatory responses, promote tissue repair, angiogenesis, and extracellular matrix remodeling, and clear apoptotic cells (a process called efferocytosis). M2 macrophages dominate the later stages of tissue repair. Importantly, M1/M2 classification represents a spectrum rather than an absolute binary dichotomy. In vivo, macrophages may show mixed phenotypes, and their phenotype can dynamically switch as environmental signals change. This plasticity is important for maintaining tissue homeostasis [15] (Table 2).

**Table 2:** Dynamic changes of M1/M2 phenotypes in cardiac repair

Phenotype	Inducing signals	Key secreted factors	Main functions
M1 type (classically activated)	• IFN-γ (from Th1 cells/NK cells) • LPS (bacterial component, mimics DAMPs signal) • GM-CSF	• Pro-inflammatory factors: TNF-α, IL-1β, IL-6, IL-12 • Effector molecules: iNOS (produces NO), ROS • Chemokines: CXCL9, CXCL10	• Pro-inflammatory response • Antibacterial/Antiviral • Clearance of pathogens and necrotic cells • Mediates tissue damage
M2a type (alternatively activated)	• IL-4, IL-13 (from Th2 cells/basophils/mast cells)	• Cytokines: IL-10, TGF-β • Characteristic enzymes: Arginase-1, Ym1/2, Fizz1 • Growth factors: IGF, PDGF	• Anti-inflammatory response • Promotes fibrosis and scar formation • Parasite clearance • Cell proliferation
M2b type (regulatory)	• Immune complexes + TLR/IL-1R agonists • IL-1R ligands	• High IL-10 • IL-1ra (IL-1 receptor antagonist) • Moderate TNF-α, IL-6	• Immunoregulation • Suppresses excessive inflammation • Promotes Th2 response
M2c type (deactivated/remodeling)	• IL-10 (from Tregs, etc.) • Glucocorticoids • TGF-β	• High TGF-β • IL-10 • MMPs (Matrix Metalloproteinases) • TIMPs (Tissue Inhibitors of Metalloproteinases) • VEGF	• Anti-inflammatory • Matrix remodeling • Phagocytosis of apoptotic cells (efferocytosis) • Promotes angiogenesis

Macrophages do not remain static during the repair process after MI, but undergo a highly dynamic and orderly phenotypic switch. The core principle is the transition from M1 dominance in the early inflammatory phase to M2 dominance in the middle and late repair phases. The timing and efficiency of this switch directly determine the repair outcome [16].

#### Phase one: acute inflammatory phase (days 1–3 post-MI) – M1 phenotype is dominant

- *Dynamic changes* DAMPs (e.g., HMGB1) released from necrotic myocardium strongly polarize resident and early infiltrating macrophages towards the M1 phenotype via TLR4 and other signaling pathways, synergizing with interferon-γ.
- *Function & necessity* M1 macrophages produce large amounts of ROS, NO, and proteases, powerfully clearing necrotic cells and matrix debris, enabling subsequent repair. This phase is a necessary “demolition” process.
- *Research data* Studies in mouse MI models have shown that on day 3 post-MI, cardiac macrophages highly express M1 markers (e.g., inducible nitric oxide synthase and IL-1β), while M2 marker (e.g., arginase 1) expression is low. IL-10-deficient mouse models show a stronger and prolonged M1 response post-MI, leading to exacerbated inflammation and worsened cardiac function, demonstrating the importance of early transition from M1 to M2.

#### Phase two: repair transition phase (days 4–7 post-MI) – M2a/M2c phenotypes begin to dominate

- *Dynamic changes* Pro-inflammatory signals in the microenvironment weaken as necrotic material is cleared. Simultaneously, regulatory T cells and other cells begin to infiltrate and secrete IL-10 and TGF-β. Additionally, increased secretion of IL-4/IL-13 by Th2 cells and related cells collectively drives macrophages towards the M2 lineage.
- *Function* The M2a type strongly activates fibroblasts via secretion of TGF-β and PDGF, promoting collagen deposition and scar formation to maintain the structural integrity of the heart. The M2c type regulates extracellular matrix remodeling via secretion of MMPs/TIMPs and promotes the resolution of inflammation by phagocytosing apoptotic neutrophils, among other mechanisms.
- *Research data* Flow cytometry analysis showed that by day 7 post-MI in mice, the proportion of macrophages expressing CD206 (general M2 marker) and arginase 1 (M2a marker) was increased. Studies using IL-4/IL-13 neutralizing antibodies or Stat6 deficient mice (key pathway for M2a polarization) showed impaired scar formation post-MI and an increased rate of cardiac rupture, demonstrating the protective role of M2a in acute phase repair.

#### Phase three: chronic remodeling phase (≥ 1 week post-MI) – M2 phenotype persistence and functional diversion

**Fig. 1:**
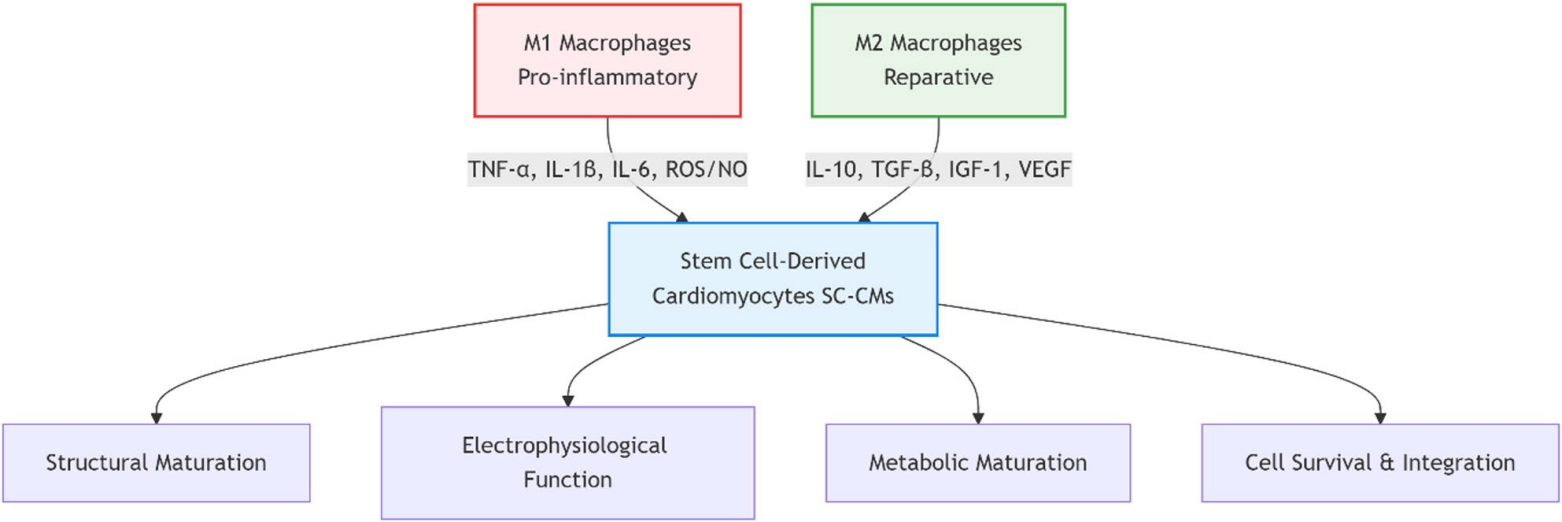
Dual regulatory effects of m1 and m2 macrophages on stem cell-derived cardiomyocyte maturation

- *Dynamic changes* Ideally, inflammation completely resolves after scar maturation. However, under pathological conditions (e.g., hypertension and diabetes) or because of a large infarct size, low-grade inflammation and abnormal repair persist.
- *Functional diversion* Regarding M2a, if the fibrotic response driven by M2a is excessive and uncontrolled, it leads to diffuse interstitial fibrosis, impairing diastolic and systolic function and promoting the progression of heart failure. Regarding the role of M2c, persistently present M2c may attempt to promote angiogenesis via VEGF secretion, but this is often insufficient to reverse ischemia (Fig. 1).
- *Research data* M2 macrophage infiltration can still be detected in the cardiac tissue of patients with heart failure and in animal models, and is closely associated with fibrotic areas. In diabetic MI models, there is delayed and impaired transition from M1 to M2, accompanied by more severe myocardial fibrosis and cardiac dysfunction. This finding indicates that dysregulation of phenotypic switching is key to a poor prognosis (Table 3).

## Dual regulatory role of M1/M2 macrophages in SC-CM differentiation and maturation

( M1 macrophages (red) secrete pro-inflammatory factors that inhibit SC-CM maturation, while M2 macrophages (green) release reparative factors that promote maturation through structural, electrophysiological, metabolic, and survival pathways.)

**Table 3:** Molecular mechanisms of macrophage-secreted factors in regulating SC-CM maturation

Macrophage type	Key factors	Effects on SC-CMs	Molecular mechanisms
M1	TNF-α	Inhibits differentiation efficiency	Inhibits Wnt/β-catenin pathway
M1	IL-1β	Impairs electrophysiological function	Disrupts calcium homeostasis, causes abnormal phosphorylation of RyR2
M1	ROS/NO	Hinders metabolic maturation	Induces mitochondrial dysfunction, maintains glycolytic state
M1	Multiple factors	Reduces cell survival	Activates death receptor and mitochondrial apoptosis pathways
M2	IL-10	Improves electrophysiological function	Enhances calcium cycling, promotes electrical synchrony
M2	TGF-β	Promotes structural maturation	Supports ECM remodeling and cell alignment
M2	IGF-1	Enhances cell survival	Activates PI3K/Akt survival pathway
M2	VEGF	Promotes angiogenesis and metabolic maturation	Provides nutritional support, improves transplantation microenvironment

Detailed analysis of specific factors, their effects, and underlying molecular pathways.

### Inhibitory effects of M1 macrophages

Numerous in vitro co-culture experiments and studies using conditioned media have shown that M1 macrophages and their key effector factors exert multi-layered inhibitory effects on differentiation and maturation of SC-CMs.

1. *Inhibition of differentiation efficiency and lineage commitment* In early differentiation, TNF-α derived from M1 macrophages inhibits activity of the Wnt/β-catenin signaling pathway, which is a key driver of cardiac lineage commitment. Studies have indicated that adding TNF-α neutralizing antibodies during the differentiation of hPSCs to cardiomyocytes increases the positive rate of cardiac-specific markers (e.g., cTnT and α-actinin), thereby improving differentiation efficiency.
2. *Impairment of structural and sarcomeric maturation* SC-CMs exposed to M1-conditioned media or an IL-1β environment show impaired development of sarcomere structure, which manifests as disorganized Z-discs and an irregular arrangement of myosin/actin. Concurrently, the expression of intercellular junction proteins, particularly the gap junction protein connexin 43 and the adhesion junction protein N-cadherin, is downregulated. This downregulation leads to impaired intercellular electrical coupling and mechanical coupling, causing difficulty in forming functional syncytia [17].
3. *Damage to electrophysiological function and calcium handling* From an electrophysiological perspective, this is the most detrimental effect of the M1 response. SC-CMs treated with M1-conditioned media show an abnormal action potential duration, reduced calcium transient amplitude, slowed kinetics, low excitation-contraction coupling efficiency, and increased susceptibility to arrhythmias [18]. The underlying mechanisms of these findings involve pro-inflammatory factors directly interfering with the expression, post-translational modification, and function of key ion channels (e.g., inward rectifier potassium channel Kir2.1 and L-type calcium channel Cav1.2) and calcium handling proteins (e.g., sarcoplasmic/endoplasmic reticulum calcium ATPase SERCA2a and ryanodine receptor RyR2). TNF-α downregulates SERCA2a expression via the nuclear factor-κB pathway, while IL-1β causes abnormal phosphorylation and calcium leakage of RyR2.
4. *Suppression of metabolic maturation* Mature cardiomyocytes primarily rely on mitochondrial fatty acid β-oxidation to produce ATP, whereas SC-CMs tend to use glycolysis, which is a fetal metabolic pattern. The M1 response hinders the metabolic maturation of SC-CMs by inducing oxidative stress and mitochondrial dysfunction. ROS produced by M1 macrophages directly damages mitochondrial DNA and electron transport chain complexes in SC-CMs, maintaining their immature glycolytic metabolic state, and thus failing to meet the high energy demands of mature myocardium [19].
5. *Induction of cell apoptosis* By activating the death receptor pathway (e.g., Fas/FasL) and the mitochondrial pathway, TNF-α and IL-1β secreted by M1 macrophages increase the apoptosis rate of SC-CMs. This pro-apoptotic effect is particularly severe in environments simulating hypoxia and inflammation post-MI, representing a major cause of massive loss of transplanted cells [20].

### Promotive effects of M2 macrophages

In contrast to M1, M2 macrophages create a favorable microenvironment for differentiation and maturation of SC-CMs by secreting various trophic factors and anti-inflammatory mediators.

1. *Promotion of differentiation and enhanced survival* M2-conditioned media upregulates the expression of various cardiac-specific genes and transcription factors (e.g., GATA4 and NKX2.5). Secreted insulin-like growth factor 1 and hepatocyte growth factor reduce apoptosis of SC-CMs by activating the PI3K/Akt pathway, a key cell survival signal, thereby inhibiting caspase-3 activity and supporting their survival [21].
2. *Support for structural maturation and alignment* TGF-β secreted by M2 macrophages, while promoting fibrosis in later disease stages under controlled in vitro conditions, contributes to mild extracellular matrix remodeling. This remodeling provides a physical scaffold and biochemical signals for the regular alignment and parallel assembly of myofibrils in SC-CMs [22].
3. *Improvement of electrophysiological functional maturation* Co-culture experiments have shown that M2 macrophages promote the electrophysiological maturation of SC-CMs. Secreted IL-10 helps improve calcium cycling function, making calcium transients more synchronous, robust, and faster to recover. Some studies have shown that SC-CMs treated with M2-conditioned media exhibit action potential morphology closer to adult cardiomyocytes, more negative maximum diastolic potential, and faster conduction velocity.
4. *Promotion of metabolic reprogramming* Factors secreted by M2 macrophages enhance mitochondrial biogenesis and function in SC-CMs, promoting the expression of peroxisome proliferator-activated receptor gamma coactivator 1-alpha and genes related to fatty acid oxidation. This process drives the metabolic shift of SC-CMs from glycolysis to oxidative phosphorylation, which is a core hallmark of functional maturation.
5. *Promotion of angiogenesis and trophic support* M2 macrophages are a major source of VEGF. In vivo, VEGF is crucial for the survival of transplanted SC-CMs. By promoting host blood vessel growth into the transplanted cell mass, M2 cells provide essential oxygen and nutrients, addressing the core bottleneck for their survival. Simultaneously, M2 cells directly support the metabolic function of SC-CMs through the secretion of various trophic factors [23] (Table 4) .

**Table 4:** *Comparison of the effects of M1 and M2 macrophages on SC-CMs*

Feature	M1 macrophages (pro-inflammatory)	M2 macrophages (reparative)
Key inducing signals	Th1 cytokines (IFN-γ, TNF-α), LPS	Th2 cytokines (IL-4, IL-13, IL-10), TGF-β
Main secreted factors	TNF-α, IL-1β, IL-6, IL-12, ROS/NO	IL-10, TGF-β, IGF-1, VEGF, HGF
Effect on differentiation	Inhibits efficiency, interferes with lineage commitment	Improves efficiency, supports progenitor cell survival
Effect on structural maturation	Disordered sarcomeres, reduced connexins (Cx43)	Promotes ECM remodeling, supports cell alignment
Effect on electrophysiology	Abnormal APD, impaired calcium handling, pro-arrhythmic	Improves calcium cycling, promotes electrical synchrony
Effect on metabolism	Maintains fetal-like glycolysis, impaired mitochondria	Promotes fatty acid oxidation metabolic maturation (OxPhos)
Effect on Cell Survival	Induces apoptosis (death receptor/mitochondrial pathways)	Promotes survival (activates PI3K/Akt pathway)
Effect on angiogenesis	Inhibits angiogenesis	Promotes angiogenesis (via VEGF)
Role in MI context	Initial response clears dead cells/ECM debris; inflammatory cytokines exacerbate damage.	Promotes repair/regeneration of damaged myocardium; secretes IL-10, TGF-β to aid repair.
Overall effect	Pro-inflammatory, Pathogen Clearance, Anti-tumor	Anti-inflammatory, Tissue Repair, Angiogenesis, Fibrosis

However, a critical analysis reveals that the M1/M2 paradigm is an oversimplification of a continuous spectrum of macrophage states. The effect of macrophage-derived signals on SC-CMs is not always binary and can be context-dependent. For instance, while TNF-α is predominantly considered detrimental, some studies suggest that its low-level or transient exposure might precondition SC-CMs and enhance their resilience against subsequent inflammatory stress [24]. Similarly, the M2 cytokine TGF-β, while promoting SC-CM alignment, can also potently induce fibrosis and hypertrophy if overexpressed or sustained, highlighting a double-edged nature that is often overlooked in in vitro maturation models [25]. Furthermore, the source of macrophages (e.g., human vs. rodent, monocyte-derived vs. tissue-resident) and the specific SC-CM maturation protocol used can lead to divergent findings across studies, making direct comparisons challenging. Acknowledging these controversies and contextual nuances is crucial for a realistic interpretation of the field and for designing more precise, effective therapeutic interventions.

## Therapeutic strategies targeting macrophage polarization and clinical application prospects

The core limitation of current mainstream treatment strategies for MI (e.g., percutaneous coronary intervention and drug therapy) is that while they can restore blood flow and alleviate symptoms, they cannot reverse an already necrotic myocardium or effectively prevent subsequent adverse ventricular remodeling and the development of heart failure. Therefore, this therapeutic dilemma led to the emergence of stem cell therapy strategies. The core advantage of hPSC-cardiomyocytes is that they not only directly replace damaged cardiomyocytes, but through powerful paracrine effects, regulate the cardiac immune microenvironment, promote angiogenesis, inhibit fibrosis, and activate endogenous repair mechanisms. This process could lead to the possibility of fundamentally repairing cardiac structure and function and reversing the progression of heart failure. Based on the above-mentioned mechanistic studies, actively regulating macrophage polarization has become an emerging frontier area for optimizing SC-CM therapeutic strategies.

### Optimization of cell therapy

1. *Timing of transplantation* Avoiding the early inflammatory peak post-MI (days 1–3) and choosing the time window when inflammation begins to subside and the repair program initiates (approximately days 5–7 post-MI) for SC-CM transplantation efficiently uses the endogenous M2 macrophage-dominated reparative microenvironment. This situation improves the survival rate of transplanted cells [26].
2. *Combined cell transplantation* Co-transplanting SC-CMs with supportive cells possessing immunomodulatory functions is a highly promising strategy. Mesenchymal stem cells (MSCs) secrete large amounts of IL-10, TGF-β, PGE2, and other factors. This secretion effectively inducing host macrophages to polarize towards the M2 phenotype, creating a local immune-privileged or immune-supportive microenvironment for the co-transplanted SC-CMs. Similarly, directly co-transplanting in vitro pre-polarized M2 macrophages also improves the outcome of SC-CM transplantation [27].

### Engineering and material science strategies

1. *Smart biomaterials* Smart hydrogels or microneedle patches that respond to the local microenvironment can be designed as delivery vehicles for SC-CMs. These materials can be loaded with cytokines, such as IL-4, IL-13, and IL-10, releasing them in a sustained and controlled manner at the injury site. This release actively “educates” infiltrating macrophages into the M2 phenotype, thereby improving the transplantation microenvironment. A research team led by Prof. Ke Cheng developed an IL-4 microneedle patch that successfully induced cardiac macrophage M2 polarization in a MI model, which promoted repair processes [28].
2. *Exosome and extracellular vesicle therapy* Exosomes derived from M2 macrophages or MSCs are rich in immunomodulatory micro RNAs (miRNAs) (e.g., miR-21, miR-146a, and miR-181) and proteins, and can serve as cell-free therapies, mimicking the beneficial effects of M2 macrophages while avoiding the complexity and potential risks of direct cell transplantation [29]. These nanoscale vesicles are easily taken up by other cells, enabling efficient intercellular communication and functional regulation.

### Other therapeutic strategies targeting macrophage polarization

Based on the critical role of macrophage polarization in cardiac disease, regulating their polarization balance has become a potential therapeutic strategy.

1. Phytochemicals and natural compounds

(i) *Resveratrol* Resveratrol promotes M2 polarization via the SIRT1/AMPK/VEGF-B axis. Clinical studies have shown that 100–400 mg of resveratrol daily reduces NT-proBNP levels in patients with heart failure. More importantly, resveratrol treatment enhances the differentiation efficiency of hPSCs into cardiomyocytes and drives SC-CMs towards a more mature phenotype, such as improving sarcomere alignment and metabolic maturation, which is crucial for their post-transplantation function [30]. Simultaneously, when using adult stem cells (e.g., MSCs) for therapy (the mechanism is primarily paracrine), this activates protective pathways in the cells, enhancing their resistance to more beneficial exosomes and growth factors, thereby amplifying their therapeutic benefits [31].
(ii) *Astragaloside IV* SC-CM transplantation faces severe challenges of an ischemic, inflammatory, and oxidative stress microenvironment. Astragaloside IV enhances the survival of transplanted SC-CMs through its potent anti-apoptotic, antioxidant [32], and anti-inflammatory properties. Moreover, it directly promotes cardiomyocyte function by stabilizing the cell membrane and improving mitochondrial fitness and calcium homeostasis. Astragaloside IV promotes stem cell proliferation and paracrine function, and stabilizes the cardiomyocyte membrane, improving mitochondrial function and regulating calcium homeostasis to achieve protection of cardiomyocyte function [33].
(iii) *Berberine* Berberine modulates the SIRT3-RKIP-TBK1 pathway. Oral administration of 1.2–2.0 g of berberine daily improves the left ventricular ejection fraction in patients with chronic heart failure [34]. Additionally, berberine possesses antioxidant and anti-endoplasmic reticulum stress properties [35], exhibits anti-inflammatory, anti-fibrotic, and pro-survival effects on stem cells, while also directly protecting and potentially maturing cardiomyocytes [36]. The combination of berberine with SC-CM therapy represents an innovative metabolic intervention–cell therapy combined strategy, allowing the success of cell therapy at systemic and local levels.

(2) *Neuromodulation* Vagus nerve stimulation or the use of its receptor agonists (e.g., acetylcholine) inhibits M1 macrophage activation and potentially promotes their shift towards the M2 type via the α7nAChR-JAK2/STAT3 signaling pathway. This shift towards the M2 type reduces the inflammatory response post-MI and protects the myocardium [37].
(3) *Cell therapy and innovative technologies* Recent studies have indicated that importing healthy exogenous mitochondria into macrophages results in mitochondrial transplantation macrophages, which autonomously polarize to an M2 phenotype. Mitochondrial transplantation macrophages show enhanced metabolic activity and repair function, not only targeting the infarcted myocardium but also indirectly improving the energy metabolism of cardiomyocytes through mitochondrial transfer. This process reduces fibrosis, promotes angiogenesis, and improves cardiac function [38].
(4) *Epigenetic regulation* Histone modifications (e.g., lactylation) regulate macrophage gene expression. In MI, histone H3K18 lactylation promotes the transcription of reparative genes in monocyte–macrophages, thereby modulating their anti-inflammatory and pro-angiogenic activity, favoring cardiac repair. Intervening in these epigenetic modifications may represent a new strategy.

## Limitations of current experimental methods and future prospects

The current research in this field still has several main limitations that need to be addressed in the future. ① The type of model used in studies is a limitation. Most studies relied on two-dimensional cell co-culture or mouse xenograft models. These models cannot fully simulate the highly complex, fibrotic three-dimensional microenvironment of human pancreatic cancer, particularly the dynamic, long-term interactions between PSCs, macrophages, and other cells (e.g., T cells and cancer cells). ② There is insufficient detail in mechanistic studies. Many studies only observed the association between M2 macrophages and PSC activation. The identification of specific signaling hubs (e.g., key cytokines, exosomes, and metabolites) and their downstream pathways involved in these two variables is still unclear, lacking direct causal evidence. ③ Previous studies neglected heterogeneity. Macrophages and PSCs are highly heterogeneous and plastic [39]. Experiments often treated these cells as single populations (e.g., M1/M2, quiescent/activated), ignoring the existence of subpopulations and their functional diversity at different tumor stages or spatial locations. This omission limits the understanding of the precision of targeted therapies. ④ There are challenges in therapeutic translation: Although targeting macrophages (e.g., using CSF-1R inhibitors) shows potential in preclinical models, the results are often unsatisfactory and may be accompanied by compensatory mechanisms. This limitation highlights the incomplete knowledge of the powerful immunosuppressive network of the tumor microenvironment and the huge challenge of translating single-target strategies into effective clinical regimens.

Future studies need to develop therapies that can reverse pro-tumor M2 to anti-tumor M1 or directly inhibit M2 polarization, combined with chemotherapy and immunotherapy, to reshape the tumor microenvironment and reduce the risk of PSC-driven pancreatic cancer progression [40].

## Challenges and future perspectives

The core role of macrophage polarization in the differentiation and maturation of SC-CMs, with the inhibitory effects of M1 and the promotive functions of M2, provide a solid theoretical basis and various strategic options for myocardial regeneration therapy. However, this research field still faces many severe challenges in moving from basic research to clinical applications.

1. *Macrophages show high heterogeneity* The traditional M1/M2 dichotomy is a simplification of their complexity. With the application of technologies, such as single-cell sequencing, researchers have discovered more functionally distinct macrophage subsets in the heart (e.g., CCR2+, LYVE1+, and TREM2+) [41]. Future research needs to more finely dissect the precise effects of these specific subsets on SC-CMs, rather than focusing broadly on M1/M2.
2. *Timing of regulation is crucial.* Inflammation and repair are a dynamic continuum. How to precisely control the timing of intervention to synchronize macrophage phenotypic switching with the tissue repair process is crucial for therapeutic success. Premature induction of M2 polarization may be detrimental to the clearance of necrotic tissue, while being too late may miss the optimal repair window. In the late phase of MI, excessive or persistent M2 responses may lead to adverse fibrosis and ventricular remodeling [42].
3. *Clinical application research still needs strengthening* Currently, most mechanistic experimental research is still conducted in two-dimensional cell cultures or mouse models. The hearts of large animals (e.g., pigs) are closer to humans in size, heart rate, and the immune system. The safety and efficacy of these immune regulation strategies need validation in such models.
4. *Long-term safety requires careful evaluation* Particularly for engineered cell therapies and gene regulation strategies, their potential tumorigenicity, immunogenicity, and off-target effects require extremely rigorous and long-term evaluation [43].
5. *Context-dependent effects of M2-associated factors* A significant challenge lies in reconciling contradictory findings regarding specific macrophage-secreted factors. For example, IL-10 is widely recognized as an anti-inflammatory and protective cytokine from M2 macrophages, promoting SC-CM calcium handling [44]. Yet, other reports indicate that sustained IL-10 signaling might inadvertently maintain a fetal-like gene program and inhibit terminal metabolic maturation by suppressing mitochondrial biogenesis [46]. This apparent paradox underscores the importance of factor concentration, timing, and cellular context, suggesting that a mere “M2 boost” may be insufficient and that a more nuanced, temporally controlled approach is required.

Based on these challenges, future research should focus on the following directions. (1) Single-cell multi-omics technologies and spatial transcriptomics need to be used to map the detailed interaction landscape between immune cells and cardiomyocytes during cardiac repair, discovering new regulatory targets. (2) “Off-the-shelf” engineered cell products, such as iPSC-derived M2 macrophages or MSCs overexpressing specific factors, need to be developed to achieve standardized production [46]. (3) Smart responsive biomaterials capable of dynamically releasing immunomodulatory factors based on local inflammatory status (e.g., pH and ROS levels) need to be designed, achieving precise drug delivery [47]. (4) Clinical trials of SC-CM transplantation combined with immune regulation need to be performed, moving from proof-of-concept to clinical reality.

In conclusion, combining myocardial regenerative medicine with immune microenvironment regulation, “taming” macrophages to pave the way for transplanted SC-CMs, represents a highly promising paradigm shift. Interdisciplinary collaboration, with the integration of cell biology, immunology, materials science, and clinical medicine, holds the potential to ultimately overcome the issues in myocardial regeneration therapy and to achieve a real breakthrough from the laboratory to the clinical setting.

## Data Availability

The datasets used and/or analysed during the current study available from the corresponding author on reasonable request.
